# Development and Preliminary Evaluation of a Pharmacogenetic Panel for Risk Stratification of Liver Injury in Polypharmacy

**DOI:** 10.3390/biomedicines14102162

**Published:** 2026-09-24

**Authors:** Iurii Slepov, Evgeniy Kopylov, Konstantin Blagodatskikh, Nadezhda Gladysheva, Dmitriy Melchenko, Karin Mirzaev, Dmitry Sychev, Roman Deev

**Affiliations:** Petrovsky Russian Scientific Center of Surgery, Moscow 119435, Russia; evgenijkopylov19540@gmail.com (E.K.); k.blag@yandex.ru (K.B.); dimasychev@mail.ru (D.S.); romdey@gmail.com (R.D.)

**Keywords:** DILI, pharmacogenomics, PCR, polymorphism, gene, hepatotoxicity

## Abstract

**Background**: Although drug-induced liver injury (DILI) remains a concern in polypharmacy, especially in older cardiovascular patients, pharmacogenetic markers are not routinely used for DILI risk prediction. Genome-wide polygenic scores aggregate thousands of variants but remain costly for routine care. In this study, we developed and preliminarily evaluated a PCR-based pharmacogenetic panel targeting key drug metabolism and transport genes for hepatotoxicity risk stratification in polypharmacy patients. **Methods**: A real-time PCR panel covering 12 polymorphisms in *NAT2*, *CYP2C9*, *UGT2B7*, *ABCB1*, and *SLCO1B1* was developed using fluorescent probe melting. Autopsy liver samples and clinical data were obtained from 47 patients over 50 years old who had taken five or more cardiovascular drugs for at least six months. Liver morphology was assessed histomorphometrically using METAVIR fibrosis/activity scores and a composite Hepatic Lesion Index (HLI), while an integrated pharmacogenetic (PGx) score combined *CYP2C9* metabolizer status, *NAT2* acetylation, and *SLCO1B1*/*OATP1B1* transport function. **Results**: Significant fibrosis (METAVIR F ≥ 2) was found in 36.2% of patients, and no liver was entirely histologically normal. Carriage of the NAT2 rs1799931 minor allele was strongly associated with a higher HLI (median 7.0 vs. 4.0; adjusted *p* = 0.00013) and was the only independent predictor of fibrosis stage in multivariable regression (OR = 16.36; *p* = 0.00268). **Conclusions:** A limited, accessible five-gene panel identified clinically relevant variability in hepatotoxicity-associated genetic profiles among polypharmacy patients, with NAT2 status showing the strongest association with liver injury severity. These preliminary findings support further validation of targeted pharmacogenetic screening as a potentially scalable and cost-effective alternative to genome-wide approaches for assessing hepatotoxicity susceptibility in vulnerable populations.

## 1. Introduction

Acute or chronic liver injury caused by medications or other dietary supplements, known as drug-induced liver injury (DILI), remains a pressing issue in modern pharmacotherapy given the increasing use of potentially hepatotoxic agents and the difficulty of early diagnosis for this condition [1]. In contrast to the dose-dependent toxicity of a number of drugs, which classically include acetaminophen, idiosyncratic dose-independent injuries are often unpredictable, are not reproduced in experimental models, and cannot be reliably predicted at the prescribing stage [2]. In the first case, with dose-dependent DILI (type A), liver damage is caused by a direct toxic effect of the drug or its metabolites, which occurs upon reaching a certain exposure (dose, concentration, or duration of administration) and is reproducible in most people, as reflected in a clearer dose–response relationship and the possibility of modeling in animals. In idiosyncratic DILI (type B), damage is associated with immune and genetically determined hypersensitivity mechanisms; the dysregulation of responses to reactive metabolites; the development of hepatocellular, cholestatic, or mixed damage at normal therapeutic doses; and a variable, often long, latency period [3].

In addition to the characteristics of the drug itself (chemical structure, lipophilicity, daily dose), clinically significant risk factors for the development of DILI include female gender, older age, increased body mass index, and the presence of underlying liver pathology, as well as polypharmacy and comorbidity, which make it difficult to promptly detect the first signs of damage [4].

The prevalence of polypharmacy among patients with chronic liver diseases exceeds 50%, and the number of concomitant medications increases significantly with age and correlates with the severity of liver damage according to the Child–Pugh scale [5,6]. In elderly patients with comorbidity and polypharmacy, DILI is often characterized by a shift towards the cholestatic phenotype and is associated with an increased frequency of adverse outcomes, including mortality not directly related to liver injury [7]. In the Spanish DILI Registry cohort, the likelihood of the cholestatic phenotype increased with age and was particularly high in patients over 85 years of age [7]. Potential drug–drug interactions are extremely common among hospitalized elderly patients [8], and age-associated changes in pharmacokinetics, drug interactions, and individual variability in genes involved in metabolism, transport, and immune response may jointly modify the risk of DILI [9]. However, current diagnostic and prognostic approaches are primarily based on clinical and laboratory parameters, the assessment of temporal relationships, and the exclusion of alternative causes. Despite proven drug-specific HLA associations, pharmacogenetic markers have not yet been integrated into validated routine models for individual DILI prediction [9,10].

Currently, increasing evidence has demonstrated the importance of genetic background in the development of DILI, understood as a set of polymorphisms in the genes related to drug metabolism, transporters, and the immune system, which modify the likelihood of toxic or immune-mediated reactions [1]. Pharmacogenetic studies have shown that variations in the genes *CYP*, *NAT2*, *GST*, and *UGT* and transporters of organic anions and bile acids (e.g., *SLCO1B1*) can increase the risk of dose-dependent and idiosyncratic DILI. These genetic variations are associated with changes in the exposure to reactive metabolites and the intrahepatic accumulation of drugs, whereas polymorphisms of HLA class I/II and other immune genes are associated primarily with idiosyncratic forms, realized as drug-induced autoimmune liver damage [11]. It has been shown that the presence of certain genotypes in a patient, such as *CYP3A53/3* or *SLCO1B11/5*, increases the likelihood of developing drug-induced liver injury during therapy with certain antibiotics. Meanwhile, HLA-B*57:01 increases the risk of DILI caused by flucloxacillin by tens of times, with a high negative predictive value for the absence of this allele [12,13].

In these conditions, integrating pharmacogenetic testing into clinical practice in patients with polypharmacy appears to be a promising approach to optimizing drug therapy and preventing adverse drug reactions. Expanded pharmacogenetic testing in patients with polypharmacy allows for the identification of clinically significant drug–gene and drug–gene–drug interactions, justifying the need for dose adjustment, drug substitution, and enhanced clinical and laboratory monitoring [14]. However, most existing panels and algorithms for personalized medicine have been developed primarily to assess the efficacy, dose selection, and prevention of known adverse reactions of individual drugs, rather than for a comprehensive stratification of the risk of hepatotoxicity during the simultaneous administration of multiple drugs [15]. This underscores the importance of developing and preliminarily validating a specialized pharmacogenetic panel, incorporating polymorphisms in the *NAT2*, *CYP2C9*, *UGT2B7*, *ABCB1*, and *SLCO1B1* genes, to stratify the risk of liver injury in the context of polypharmacy, thereby enabling a shift from universal guidelines to a personalized assessment of hepatotoxicity risk in the most vulnerable patient populations.

## 2. Materials and Methods

Our study included autopsy liver tissue samples from patients over 50 years of age with a history of taking five or more medications for six or more months for the treatment of cardiovascular diseases. For each patient, individual drugs and duration of exposure were extracted from medical records. The most frequently prescribed drugs included beta-blockers (bisoprolol, metoprolol), mineralocorticoid receptor antagonists (spironolactone), statins (atorvastatin, rosuvastatin), anticoagulants (warfarin, rivaroxaban), loop diuretics (furosemide, torasemide), and antiplatelet agents (acetylsalicylic acid, clopidogrel). DILI severity was graded according to DILIN criteria based on a set of serum ALT, AST, ALP, bilirubin, and INR values, obtained during the patients’ final hospitalization before death (duration range: 2 weeks to 1.5 months) (Supplementary File S1). Biochemical data were obtained from two consecutive blood tests and analyzed retrospectively. Alcohol consumption and smoking were not exclusion criteria. Patients with cancer and hepatitis of any etiology were excluded from the study. Cases with significant autolytic changes related to the timing of the autopsy were also excluded.

### 2.1. Panel Development and Pharmacogenetic Risk Assessment

A limited set of genes responsible for key stages of hepatic drug biotransformation and transport was used to develop the pharmacogenetic panel. Based on an analysis of the literature, genes mediating Phase I and Phase II xenobiotic metabolism, as well as drug transport across hepatocyte membranes, were selected.

The integrated pharmacogenetic (PGx) score (range 0–6) was calculated as the sum of dysfunction scores (0–2 points each) across three functionally independent axes: CYP2C9 metabolism (based on the CPIC activity score), NAT2 acetylation, and OATP1B1 (SLCO1B1) transport. Three SLCO1B1 loci (rs55901008, rs56061388, rs72559745) were monomorphic across the cohort and were excluded from score calculation, as they did not contribute discriminating information; the OATP1B1 axis was therefore derived from the remaining polymorphic SLCO1B1 variant (rs4149056).

### 2.2. PCR Genotyping Technique

Patient genotyping was performed using real-time PCR, followed by melting of fluorescent probes. The BioMaster PCR-RV reagent kit (2×) (Biolabmix, Novosibirsk, Russia) and specific primers with probes (Supplementary File S2) were used. A key feature of the reaction was that the first step (real-time PCR) detected a genotype-independent signal (PCR control), followed by melting of the probe specific to the mutant allele. The melting peak for homozygous mutations was higher than for homozygous wild-type mutations, while heterozygotes exhibited two peaks.

The reaction was carried out on a BioRad CFX96 instrument (Bio-Rad, Hercules, CA, USA) using a program of 95 °C for 3 min (95 °C for 15 s—60 °C for 40 s × 40 cycles) + melting from 30 °C to 95 °C in 1 °C increments. To accumulate the matrix for melting the probes, asymmetric PCR was used with a primer ratio of 1 to 4 (forward or reverse in excess, depending on the chain to which the probe is hybridized).

### 2.3. Histomorphometric Study of Autopsy Material

Biopsies were fixed in 10% neutral-buffered formalin, passed through increasing concentrations of alcohol, and embedded in paraffin. Sections of 3 µm thickness were prepared using an MT1 rotary microtome (Dakewe, Shenzhen, China).

After deparaffinization, sections were stained with hematoxylin and eosin according to Mallory, and digital images were obtained using a KFBio KF-PRO-120 histological slide scanner (KFBio, Yuyao, China). Aperio ImageScope software (version 12.4.6.5003) was used for the analysis of liver tissue histotopograms and for morphometric assessment. Liver fibrosis and hepatocyte necosis were assessed using the METAVIR scale. The type of inflammatory infiltration was determined relative to the portal tracts, and the severity of inflammatory infiltration was calculated based on the number of involved liver acinar zones. The type of steatosis, the type of cholestasis, and the number of apoptotic bodies and Mallory–Denk bodies per mm^2^ were also assessed.

To provide an integrated assessment of hepatic injury severity, we calculated the Hepatic Lesion Index (HLI) as the unweighted sum of four morphometric components: METAVIR fibrosis stage (F, range 0–4), METAVIR necroinflammatory activity grade (A, range 0–3), extent of immune infiltration (range 0–3) and severity of cholestasis (range 0–2). The resulting HLI ranged from 0 to 12.

### 2.4. Sample Characteristics

The study included 49 patients followed at a single Moscow center, with anamnestic, clinical and laboratory data and paraffin-embedded liver tissue samples collected for each patient. Two patients were unsuitable for morphometric analysis and were therefore excluded from further analysis. The clinical and anamnestic data for the 47 patients are presented in Table 1.

### 2.5. Statistical Analysis

Statistical analysis was performed using R (version 4.x, R Foundation for Statistical Computing, Vienna, Austria). Continuous variables were summarized as medians with interquartile ranges [Q1; Q3] given the small sample size and non-normal distribution of most morphometric parameters, while categorical variables were summarized as absolute and relative frequencies. Proportions of morphological findings (significant fibrosis, high hepatitis activity, cholestasis, steatosis) were reported with 95% Wilson score confidence intervals.

Pairwise comparisons between genotype- or phenotype-defined groups were performed using the Brunner–Munzel test. For each comparison, the stochastic superiority effect size ($\hat{p}$) was reported alongside the asymptotic *p*-value. Given the small sample sizes, exact or Monte Carlo permutation-based *p*-values (based on resampling of group labels, ≥10,000 permutations) were additionally computed and reported alongside the asymptotic *p*-values. *p*-values from these pairwise comparisons were adjusted for multiple comparisons using the Benjamini–Hochberg false discovery rate procedure, applied separately within each morphological endpoint: for each endpoint, the comparison family comprised all 15 genetic predictors tested against it (12 individual polymorphisms plus three composite pharmacogenetic phenotypes—*NAT2* slow-acetylator status, *CYP2C9* IM/PM, and ≥2 pharmacogenetic risk factors).

### 2.6. Ethics

The study was conducted in accordance with the Declaration of Helsinki and was approved by the Institutional Ethics Committee of Petrovsky National Research Center of Surgery (protocol No. 1 approval date: 23 January 2026).

## 3. Results

### 3.1. PCR Panel

The panel included 12 gene polymorphisms—*NAT2*, *CYP2C9*, *UGT2B7*, *ABCB1*, and *SLCO1B1*—covering phases I and II of xenobiotic metabolism, as well as drug transport across hepatocyte membranes. The panel composition is presented in Table 2.

The selected primers were validated using control plasmids containing the mutant allele and control DNA samples previously obtained from whole-exome studies. All test systems yielded valid results for the control samples.

Morphometric analysis revealed significant fibrosis (METAVIR F ≥ 2) in 17 patients (36.2%; 23.1–51.5) (Figure 1A), high hepatitis activity (METAVIR A ≥ 1) in 27 (57.4%; 42.3–71.4) (Figure 1B), cholestasis of any grade in 21 (44.7%; 30.5–59.8) (Figure 1C), and steatosis (macro- and/or microvesicular) in 12 (25.5%; 14.4–40.6) (Figure 1D). The median HLI for the cohort was 5.0 (Q1–Q3: 2.5–6.0), with an observed range of 1–9. In no instance was the liver completely intact (HLI ≥ 1 in all cases). Statistical characteristics of the morphometric parameters are presented in Supplementary File S3.

In a PCR study of samples from 47 included patients, three *SLCO1B1* loci (rs55901008, rs56061388, rs72559745) were found to be monomorphic: all 47 samples had a wild-type genotype, and the minor allele was not detected. The absence of minor alleles at these three loci is consistent with their reported low population frequencies rather than genotyping error, as all analyzed loci—including the monomorphic *SLCO1B1* variants—showed allele frequencies concordant with reference population databases [16]. The remaining nine loci were polymorphic (Figure 2). For *NAT2* rs1799931, no homozygotes for the minor allele were detected. The results for most genotypes were consistent with Hardy–Weinberg equilibrium. Deviations were observed for *NAT2* rs1041983 and *CYP2C9* rs1799853 (*p* < 0.05), which may reflect the small sample size and the resulting wide confidence intervals rather than genotyping error; nevertheless, this should be considered a limitation (Supplementary File S4).

Based on the diplotypes of *CYP2C9* (rs1799853 and rs1057910) with calculation of the activity score according to CPIC criteria, normal metabolism was predicted in 29 patients (61.7%), intermediate metabolism was predicted in 16 (34.0%), and slow metabolism was predicted in two (4.3%); the median activity score was 2.0 (Q1–Q3: 1.5–2.0). For the integrated assessment of pharmacogenetic risk, the total panel score (0–6) was calculated as the sum of disorders along three functionally characterized axes: *CYP2C9* metabolism, *NAT2* acetylation, and *OATP1B1* transport. The median was 1.0 (Q1–Q3: 0.5–2.0); high pharmacogenetic risk (≥2 points) was found in 16 patients (34.0%), while a completely favorable profile (0 points) was found in only 10 (21.3%).

### 3.2. Associations of Panel Genotypes with Morphological Manifestations of Hepatotoxicity

An association was found between the *NAT2* rs1799931 (ww = 39 (83.0%), wh = 8 (17.0%), mm = 0 (0%)) polymorphism and the integrated severity of liver disease. Carriers of the minor allele (*n* = 7) had a higher HLI than wild-type homozygotes (*n* = 39): median 7.0 vs. 4.0 (*p* = 0.000011; $\hat{p}$ = 0.824; adjusted *p*-value = 0.00013; Monte Carlo *p*-value = 0.0045) (Figure 3A). The same locus was associated with fibrosis, but statistical significance disappeared after correction for multiple comparisons (median 3.0 vs. 1.0; *p* = 0.036; $\hat{p}$ = 0.797; adjusted *p*-value = 0.2; Monte Carlo *p*-value = 0.0063) (Figure 3B). When analyzing the associations of the remaining polymorphisms in the panel with morphological manifestations, several relationships were identified, though statistical significance was not achieved after introducing a correction for multiple comparisons (Supplementary File S5).

In the functional classification of patients with predicted intermediate or poor metabolizer *CYP2C9* (IM/PM, *n* = 18), hepatitis activity according to the METAVIR A scale was higher than in normal metabolizers. However, the differences did not reach statistical significance when adjusted for multiple comparisons (median 1.5 vs. 0; Brunner–Munzel test *p* = 0.026; $\hat{p}$ = 0.689; adjusted *p*-value = 0.3; Monte Carlo *p*-value = 0.021) (Figure 3C).

High pharmacogenetic risk (≥2 points) was nominally associated with more severe METAVIR F fibrosis (median 1.5 vs. 0; Brunner–Munzel test *p* = 0.025, $\hat{p}$ = 0.688; Monte Carlo *p* = 0.032), but this association did not remain significant after correction for multiple comparisons (BH-adjusted *p* = 0.2). While the PGx score showed weak-to-moderate correlations with the integral HLI (ρ = 0.329; *p* = 0.024), METAVIR F fibrosis (ρ = 0.313; *p* = 0.032), and the severity of cholestasis (ρ = 0.294; *p* = 0.045) (Figure 3D), none of these associations remained statistically significant after Benjamini–Hochberg correction for multiple comparisons (13 tests; q ≈ 0.195 for all three).

In the ordinal logistic regression with proportional odds for METAVIR F adjusted for age, gender, number of drugs, and truncated PGx score (excluding the NAT2 component to avoid structural collinearity), the only independent predictor associated with a higher stage of fibrosis was the carriage of *NAT2* rs1799931: OR = 13.09 (CI: 2.872–118.0; *p*-value = 0.00268).

## 4. Discussion

Genetic factors have a significant impact on the development of drug-induced liver injury. A number of studies have shown that variants of genes encoding drug-metabolizing enzymes, as well as certain HLA alleles, are associated with an increased risk of DILI in various patient populations [17,18]. However, the analysis of individual SNVs, considered in isolation, is often insufficient for predicting the occurrence of liver damage accurately. In real-life clinical practice, the risk of DILI is determined by the multifaceted interactions of a large number of genetic variants with each other and with environmental factors.

In this context, the transition from assessing single markers to modeling complex genetic interactions is logical. In particular, machine learning methods are being developed that allow for the selection of adequate statistical approaches for analyzing gene–gene and gene–environment interactions, thereby increasing the predictive value of genetic data regarding predisposition to DILI [19,20]. Large studies in this area aggregate the effects of thousands of SNVs to form polygenic risk models. For example, a genome-wide polygenic risk score for DILI (DILI PRS) has been proposed, constructed by summing the effects of more than 20,000 common variants identified in several genome-wide association studies (GWAS), going beyond the assessment of HLA alleles alone [21].

Cholestatic and mixed DILI variants have been shown to be characterized by a distinct polygenic architecture that can be quantified using PRS (CM-PRSgw). This score predicts DILI vulnerability in independent clinical cohorts (TAK-875, flucloxacillin, amoxicillin/clavulanate) and is reproducible at the level of primary hepatocytes and iPSC organoids through specific transcriptomic signatures of mitochondrial dysfunction, UPR activation, and oxidative stress [21]. From a mechanistic perspective, this score reflects a polygenic modulation of hepatocyte stress pathways (oxidative stress, endoplasmic reticulum stress, protein folding abnormalities), i.e., an intrinsic, genetically determined vulnerability of hepatocytes, and not just the presence of individual drug-specific immune triggers.

Taken together, these data highlight the need to consider the combination of multiple genetic variants in key xenobiotic metabolism and cellular stress pathways to predict DILI risk more accurately, rather than relying solely on SNV analysis. This multigene approach underlies the development of gene panels and polygenic risk scores aimed at prospectively stratifying patients based on their likelihood of developing DILI [21].

From a practical perspective, however, a full-fledged genome-wide approach based on tens of thousands of variants remains expensive and technologically challenging for widespread implementation in routine clinical practice. In resource-limited settings, a more realistic strategy appears to be focusing on a few key genes critical for drug metabolism and transport and determining a significant portion of the interindividual variability in DILI risk. A panel including *NAT2* (general metabolism), *CYP2C9*/*CYP2C19* (warfarin, torasemide), *UGT2B7* (ccarvedilol, labetalol, fluvastatin, spironolactone—inhibitor), ABCB1 (bisoprolol, dabigatran and others, spironolactone—activator), and *SLCO1B1* (statins) polymorphisms represents a functional “slice” of the polygenic architecture: it covers the main pathways of xenobiotic biotransformation and hepatic clearance, while remaining technologically accessible for PCR diagnostics and easily scalable when expanding screening to larger cohorts [1,22]. The identification of a limited number of carefully selected markers allows for a balance to be achieved between the complexity of the genetic model, the cost of testing, and the possibility of integrating pharmacogenetic screening into standard patient care pathways, especially with expected polypharmacy and the prescription of potentially hepatotoxic drugs.

The association between *NAT2* rs1799931 carriage and integrated liver injury severity (HLI) and the trend toward a link with fibrosis obtained in the present study are consistent with the previously described role of the slow acetylation of *NAT2* as a risk factor for drug-induced hepatotoxicity. In meta-analyses devoted to anti-tuberculosis drugs, the slow acetylator genotype *NAT2* has consistently been shown to increase the risk of DILI, with odds ratios ranging from 2.8 to 8.8 depending on the population and phenotype definition [23]. Furthermore, ultraslow acetylators of *NAT2* are characterized by an even higher risk compared to conventional slow acetylators, which emphasizes the clinical significance of precise phenotypic stratification by this gene and not just a binary “slow/fast” division [24]. The data obtained in our sample complement this evidence base for polypharmacy with cardiovascular drugs, and not just in anti-tuberculosis therapy, which expands the range of clinical contexts in which *NAT2* status may have prognostic value.

The observed trend towards higher hepatitis activity in CYP2C9 intermediate/poor metabolizers (IM/PM) is also consistent with data on the accumulation of hepatotoxic metabolites and reactive compounds with reduced activity of this enzyme, shown for a number of CYP2C9 substrates, including nonsteroidal anti-inflammatory drugs and anticoagulants [25]. Given the widespread use of vitamin K antagonists and NSAIDs in the study cohort, a CYP2C9-mediated reduction in clearance may be one of the mechanisms involved in the formation of a mixed hepatocellular–cholestatic pattern of injury observed in patients with polypharmacy.

In addition, the integral PGx score used in this study is fundamentally different from genome-wide polygenic risk scores (PRSs) for drug-induced liver injury, which aggregate the effects of tens of thousands of variants [21]. Such a simplified summary index is inevitably inferior in prognostic accuracy to full-fledged PRSs, but the literature indicates that the clinical utility of PRS approaches in pharmacogenetics remains limited even for large cohorts due to small sizes of training samples, heterogeneity of drug response phenotyping, and ethnic bias in effects [26,27]. To confirm the clinical significance of the PGx score, validation in a larger, independent cohort with a prospective design, including a standardized phenotypic assessment of drug response, is required.

It is worth noting that the majority of associations identified in this study lost statistical significance after adjustment for multiple comparisons; this underscores the need for a study involving a larger patient cohort. Other limitations of the present study include the pilot sample size (*n* = 47), which provides power to detect only strong effects, the use of autopsy material, and the cross-sectional nature of the study and the high prevalence of comorbidity and polypharmacy in the study population preclude establishing a causal relationship between drug exposure and the observed hepatic morphological changes. Furthermore, the regression model identifying NAT2 rs1799931 as an independent predictor of fibrosis stage was derived and evaluated in the same discovery cohort; before any clinical application, this model requires validation in an independent, adequately powered cohort to confirm the stability of the effect size and to exclude overfitting inherent to small-sample regression analyses. Similarly, the HLI, used here as an integrated measure of hepatic injury severity, has not been validated against established histological indices or in an independent cohort, and its equal-weighting scheme reflects a pragmatic choice rather than an empirically derived one. Taken together, these limitations underscore the hypothesis-generating nature of the present findings and the need for confirmatory studies in larger, independent cohorts before either the regression model or the HLI can be considered for broader clinical or research application.

## 5. Conclusions

Our preliminary findings suggest that a targeted, PCR-based panel may represent a scalable and cost-effective alternative to genome-wide polygenic scores for identifying genetic associations with hepatotoxicity in the context of polypharmacy. However, given the small sample size and cross-sectional autopsy design, these results should be regarded as hypothesis-generating. The panel’s potential utility for risk stratification and prediction of DILI in clinical practice remains to be established. Validation in larger, prospective cohorts with appropriate control groups is warranted before any clinical application can be considered.

## Figures and Tables

**Figure 1:**
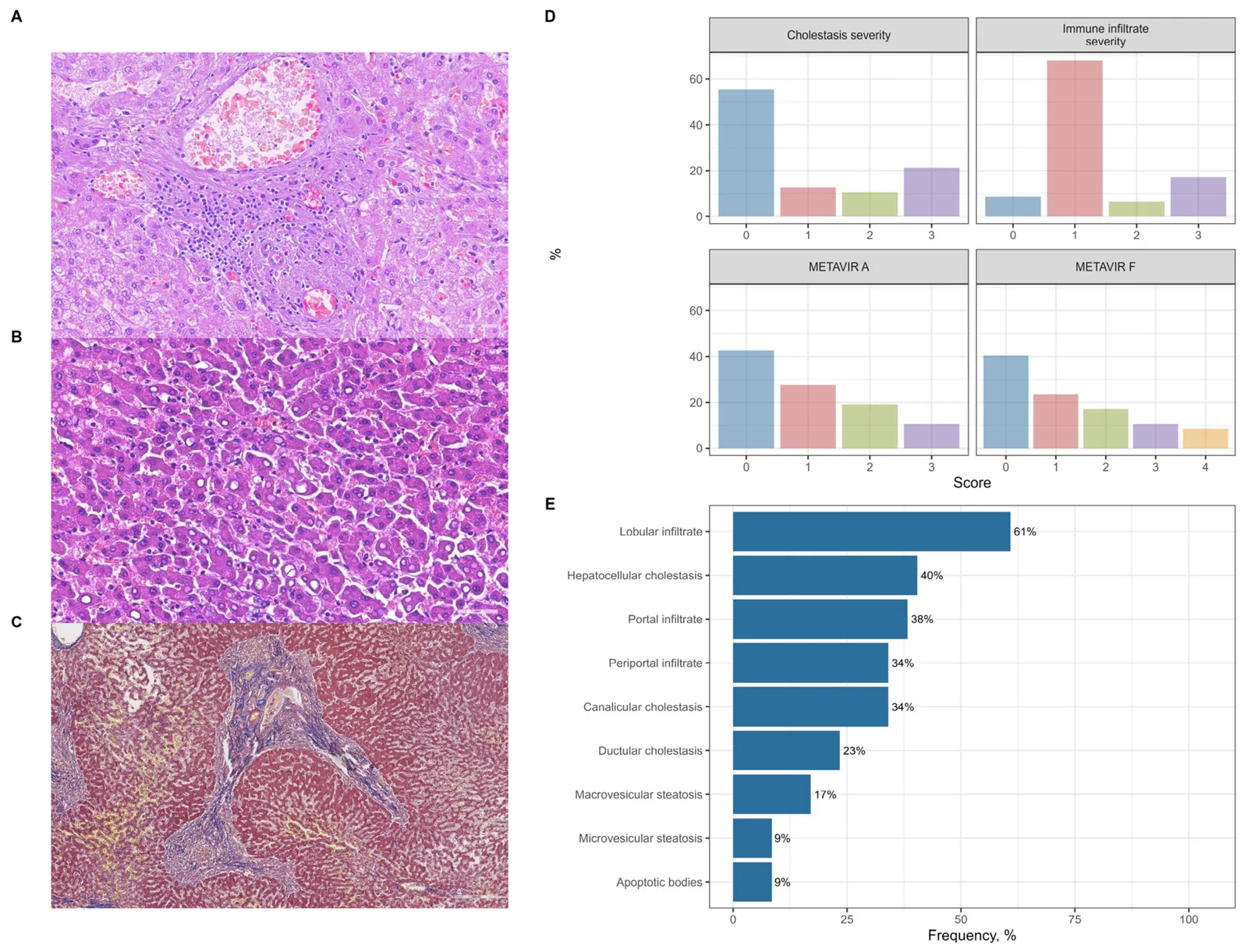
(**A**)—Periportal lymphocytic infiltration, H&E stain, ×400; (**B**)—microvesicular steatosis, H&E stain, ×400; (**C**)—bridging fibrosis (porto-portal septa), Picro-Mallory stain, ×60; (**D**)—distribution of values of semi-quantitative morphometric indicators; (**E**)—distribution of values of categorical morphometric indicators.

**Figure 2:**
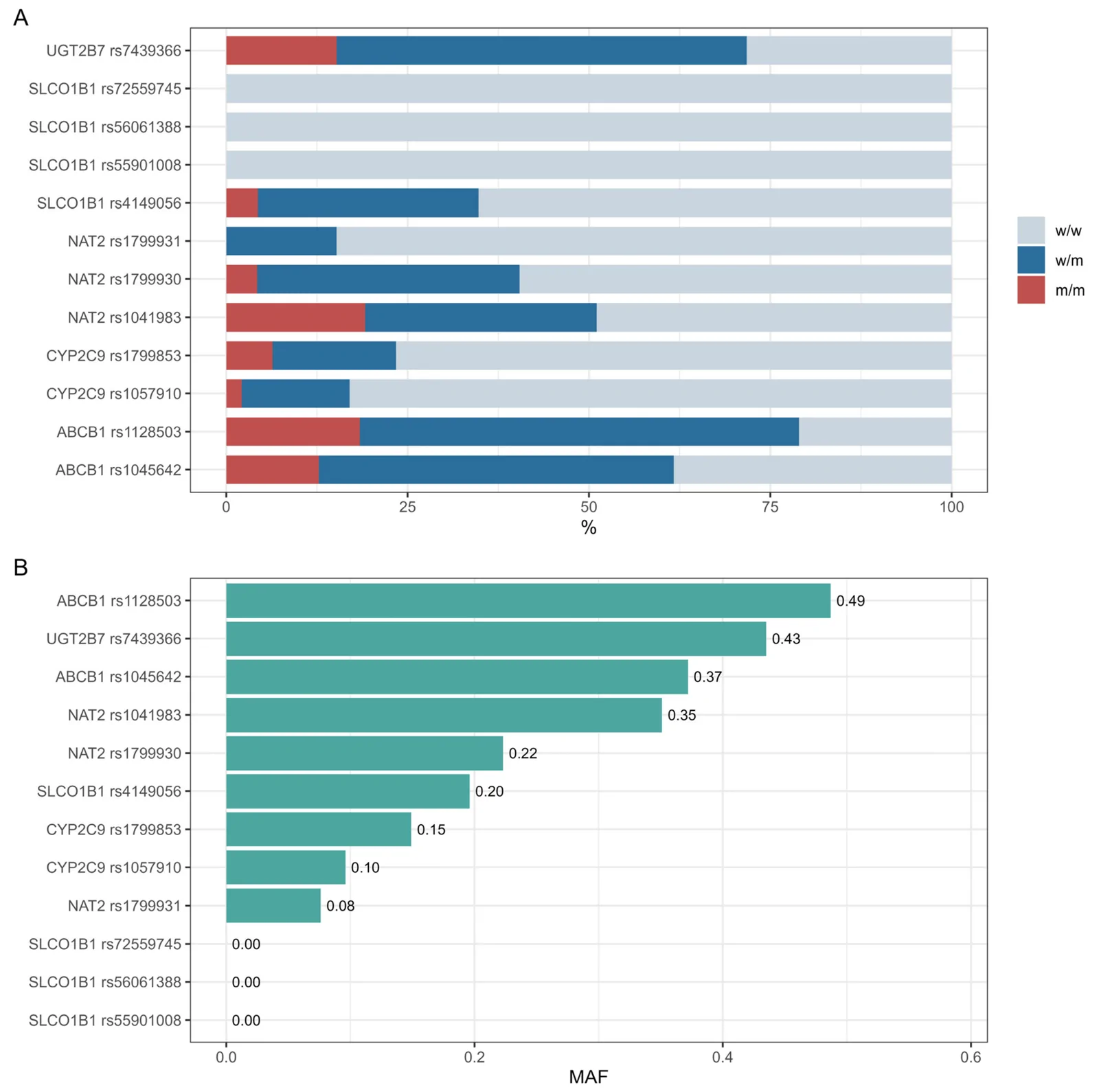
Distribution of genotypes and frequency of minor alleles of polymorphisms in the studied sample. (**A**)—Stacked bar chart showing the proportion (%) of wild-type homozygotes (w/w), heterozygotes (w/m), and mutant homozygotes (m/m) for each of the 12 studied. (**B**)—Minor allele frequencies (MAF) of the same 12 polymorphisms in the studied sample, ranked from highest to lowest; numeric MAF values are indicated next to each bar.

**Figure 3:**
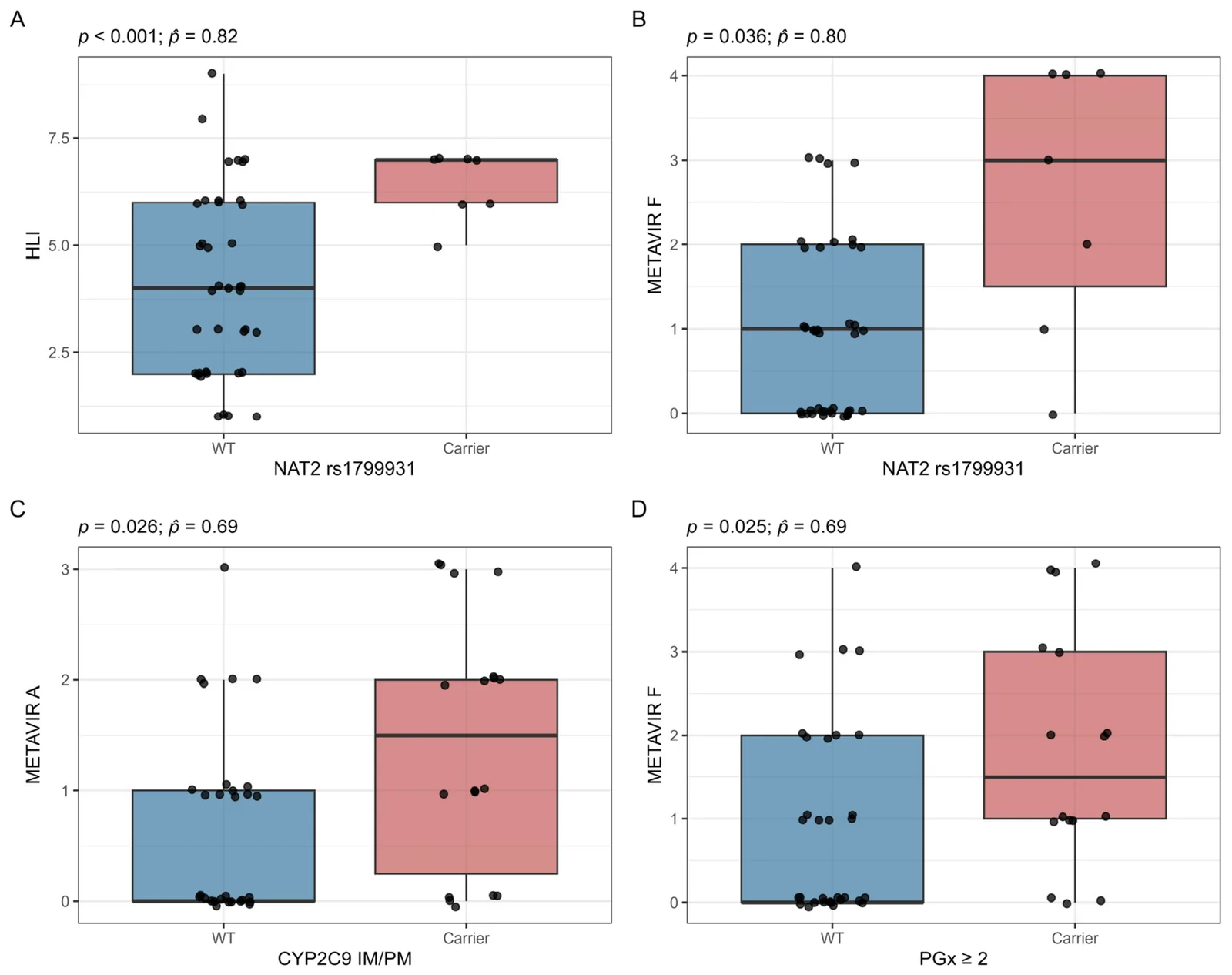
The most pronounced genotype–morphological phenotype associations. (**A**)—HLI score in wild-type (WT) versus carrier subjects for *NAT2* rs1799931. (**B**)—METAVIR fibrosis stage (F) in WT versus carrier subjects for *NAT2* rs1799931. (**C**)—METAVIR activity grade (**A**) in WT versus carrier subjects stratified by *CYP2C9* IM/PM status. (**D**)—METAVIR fibrosis stage (F) in WT versus subjects with ≥2 pharmacogenetic risk factors (PGx ≥ 2).

**Table 1:** Clinical and anamnestic data of patients.

Indicator	Meaning
Age, years, median [Q1; Q3]	77 [74; 85]
Gender: male/female	17 (36.17%)/30 (63.83%)
Main diagnosis	
Forms of coronary heart disease	27 (57.4%)
Stroke	14 (29.8%)
Valvular heart disease	4 (8.5%)
Other cardiovascular pathology	2 (4.3%)
Comorbidity	
Widespread atherosclerosis	33 (70.2%)
Hypertension	30 (63.8%)
Chronic kidney disease	25 (53.2%)
Type 2 diabetes	9 (19.1%)
Number of drugs, median [Q1; Q3]	6 [5; 7]
Classes of drugs	
Beta-blockers	39 (83%)
Mineralocorticoid receptor antagonists	26 (55.3%)
Statins	26 (55.3%)
Anticoagulants	25 (53.2%)
Loop diuretics	24 (51.1%)
DILI severity	
Mild	36 (76.6%)
Moderate	9 (19.1%)
Severe	2 (4.3%)

**Table 2:** Genes and polymorphisms.

Gene	Function	Variants (rs-ID)
*NAT2*	Acetylation of drugs (isoniazid)	rs1041983, rs1799930, rs1799931
*CYP2C9*	Metabolism of warfarin, phenytoin	rs1799853, rs1057910
*UGT2B7*	Glucuronidation of drugs	rs7439366
*ABCB1*	Transport of drugs from hepatocytes	rs1045642, rs1128503
*SLCO1B1*	Liver anion uptake and risk of statin toxicity	rs4149056, rs72559745, rs56061388, rs55901008

## Data Availability

The data presented in this study are available on request from the corresponding authors. The data are not publicly available due to privacy.

## Supplementary Materials

The following supporting information can be downloaded at: https://www.mdpi.com/article/10.3390/biomedicines14102162/s1.

## Abbreviations

The following abbreviations are used in this manuscript:

DILI	Drug-induced liver injury
NAT2	N-acetyltransferase 2
CYP2C9	Cytochrome P450 2C9
CYP2C19	Cytochrome P450 2C19
CYP3A5	Cytochrome P450 3A5
UGT2B7	UDP-glucuronosyltransferase 2B7
ABCB1	ATP-binding cassette subfamily B member 1 (P-glycoprotein gene)
SLCO1B1	Solute carrier organic anion transporter family member 1B1
OATP1B1	Organic anion-transporting polypeptide 1B1
GST	Glutathione S-transferase
HLA	Human leukocyte antigen
SNV	Single-nucleotide variant
PCR	Polymerase chain reaction
PGx	Pharmacogenetic (score)
HLI	Hepatic Lesion Index
METAVIR	Fibrosis/activity histological scoring system
CPIC	Clinical Pharmacogenetics Implementation Consortium
IM/PM	Intermediate/poor metabolizer
OR	Odds ratio
GWAS	Genome-wide association study
PRS	Polygenic risk score
CM-PRSgw	Cholestatic/mixed genome-wide polygenic risk score
iPSC	Induced pluripotent stem cell
UPR	Unfolded protein response
NSAID	Nonsteroidal anti-inflammatory drug
H&E	Hematoxylin and eosin (stain)
